# Virulence, bacterocin genes and antibacterial susceptibility in *Enterococcus faecalis* strains isolated from water wells for human consumption

**DOI:** 10.1186/2193-1801-2-43

**Published:** 2013-02-09

**Authors:** Carlos Padilla, Olga Lobos

**Affiliations:** Departamento de Microbiología, Universidad de Talca, Talca Camino Lircay s/n, Talca, Chile

**Keywords:** *E. faecalis*, Wells, Water, Bacteriocin, Virulence, Antimicrobials

## Abstract

The objectives of this study were to detect genes for virulence and bacteriocins in addition to studying the antimicrobial susceptibility of 78 strains of *E. faecalis* isolated from water wells for human consumption. The virulence and bacteriocin genes of 78 *E. faecalis* were amplified by PCR and visualized in agarose gels. The antimicrobial susceptibility was determined through diffusion agar tests and the MIC through microdilution. It was observed that the major percentage of virulence genes in the *E. faecalis* strains corresponds to *aggA* (93.5%). The bacteriocin gene *entA* (64.1%) is the most frequently detected. The studied strains exhibited different virulence and bacteriocin genes, and an important antibacterial resistance. The most common resistant phenotype (n = 14) corresponds to tetracycline and chloramphenicol and the less frequent (n = 2) to ciprofloxacin and moxifloxacin. Eight different genetic profiles were observed for virulence y bacteriocin genes. It was determined a statistical association between the bacterial resistance and some of the genetic profiles detected.

## Introduction

Representants of the genus *Enterococcus* are part of the normal human gut microbiota, some animals, and birds. The *Enterococcus* out of the organism can survive in different temperature ranges, salinity, pH and resist some detergents as dodecyl-sodium sulfate and bile (Shepard and Gilmore 2000). Phylogenetically the enterococci are divided into 7 clusters resulting finally in 33 species (Naser *et al.*^2005^; Köhler 2007). In human infections, *E. faecalis* and *E. faecium* are the most prevailing species (>90%). The first is the most commonly isolated, but recently *E. faecium* has exhibited an important increase as infectious agent (Treitman *et al.*^2005^). The enterococcal infection includes surgical wounds, endocarditis, hepatobiliary sepsis, urinary tract infections, bacteremias and neonatal sepsis among the most important (Poh *et al.*^2006^). The majority of the enterococcal infections are endogeneous, but the crossed infection occurs mainly in hospitalized patients (Cookson *et al*. 2006). *Enterococcus* in USA is the fourth most common cause of nosocomial infection and the third of bacteremia (Biendenbach *et al.*^2004^). In Europe these infections are less and represent a 7.2% of the total Martínez-Odrizola *et al.* (2007).

Enterococcal strains resistant to different antibiotics are a great public health problem, especially in isolated strains from hospital-acquired infections (Hidron et al. 2008). Several genetic studies have described the complex functions related to horizontal gene transfer in enterococcal strains, which partly explains their high antimicrobial resistance (Arthur *et al.*^1993^; Clewell *et al.*^1995^). Vancomycin was the antimicrobial agent most active against this bacterium, however, today in different parts of the world, isolated enterococcal strains are resistant to vancomycin (Ridwan et al. 2002; Chang *et al.*^2010^).

Various factors determine the *E. faecalis* virulence*,* outstanding among those on the cellular surface, as the aggregation substance (Agg), the extracellular surface protein (Esp) and some that are excreted out of the bacterial cell as cytolysin and hyaluronidase, among others (Fisher and Phillips 2009; Semedo *et al.*^2003^; Koch et al. 2004). Additionally, the *Enterococcus* spp. synthesizes heterogeneous antibacterial peptides or bacteriocins, also called enterocins (Giraffa 1995). These products may give these bacteria an additional ecological tool to persist in environments colonized by competing microorganisms in order to remove them and take over the ecological niche (Padilla et al. 2006).

In recent years, *E. faecalis* has a greater ability to cause infections on different human epithelia (Fisher and Phillips 2009). Thus, more studies are needed to provide new knowledge about this bacterial specie

Currently, there is considerable information regarding the antimicrobial susceptibility, presence of virulence genes and bacteriocins in *E. faecalis* strains isolated from clinical specimens and food. However, it would also be important to study if strains that come from water wells possess the properties mentioned and if it is possible to transmit these characteristics to the intestinal microbiota of people who drink this water.

Is important to remark that in some rural areas of central Chile, where there is no drinking water, people use well water for drinking and for various domestic purposes.

For a better understanding of the biology of *E. faecalis*, the objectives of this study were to detect genes for virulence and bacteriocins in addition to studying the antimicrobial susceptibility of 78 strains of *E. faecalis* isolated from water wells for human consumption.

## Materials and methods

### Isolates and bacterial identification

Were studied 128 water wells. In 78 of them were isolated 78 *E. faecalis* strains in the Maule Region in central Chile during the year 2010. From each well were taken 20 ml of water with a sterile bottle at a deep of about 5 cm. The samples were taken within 2 h to the microbiology laboratory of the University of Talca where they were processed immediately. The water samples were diluted 1:9 in sterile distilled water and 50 μl of this dilution was plated on Bile-esculin agar (Merck, Darmstadt, Germany) and incubated at 37°C for 24 h. The identification of the 78 bacterial isolates was carried out through conventional biochemical tests (Facklam and Collins 1989).

### Antibacterial susceptibility and determination of the minimum inhibitory concentration (MIC)

The antibacterial susceptibility test was performed through the diffusion in agar test (Bauer et al. 1996). The antimicrobials analyzed were: ampicillin, tetracycline, ciprofloxacin, moxifloxacin, gentamicin, vancomycin, chloramphenicol y erythromycin (VALTEK, Santiago, Chile). For determination of MIC, the antimicrobial were prepared in accordance with the manufacturers’ instructions (Sigma, St Louis, MO, USA) and in accordance with the recommended by EUCAST (2012). All the resolutions of the MICs were performed in duplicate. *E. faecalis* strain ATCC 29212 was included as control in assays with or without antimicrobials.

### Genes amplification and conditions for the PCR

The total DNA of *E. faecalis* strains was used in the PCR reactions to detect the presence of virulence genes as cytolysin (*cylA)*, aggregation substance *(aggA*), antigen A *(efaA),* stimulating of pheromones expression (*eep*), gelatinase (*gelE*) and surface protein (*esp*). Additionally were amplified the genes for enterocins AS-48 (*as-48*), bacteriocin 31 *(bac31*), enterocins A, B and P (*entA, entB, entP*), enterocin L50 A/B *(entL50A/B*), enterolysin A (*enlA*) and enterocin 1071 (*ent1071*). Each PCR reaction contained 10 mM Tris–HCl buffer pH 8.3, 1.5 mM MgCl_2,_ 50 mM KCl, 200 μM of each deoxinucleotide (Omega, BioTek, Doraville, GA), 1 μM of oligonucleotide, 2U of *Taq* DNA polymerase and 15 ng of DNA template (Table 1).

**Table 1:** **Oligonucleotide sequences used in this study and their products**

Virulence genes	Oligonucleotide sequences	Product size (bp)	Reference
*cylA*	for5′-TGGATGATAGTGATAGGAAGT-3′	517	Eaton and Gasson 2001
	rev5′-TCTACAGTAAATCTTTCGTCA-3′		
*aggA*	for5′-AAGAAAAAGTAGACCAAC-3′	1553	Eaton and Gasson 2001
	rev5′-AACGGCAAGACAAGTAAATA-3′		
*efaA*	for5′-GACAGACCCTCACGAATA-3′	705	Eaton and Gasson 2001
	rev5′-AGTTCATCATGCTGCTGTAGTA-3′		
*eep*	for5′-GAGCGGGTATTTTAGTTCGT-3′	937	Bittencourt de Marques and Suzart 2004
	rev5′-TACTCCAGCATTGGATGCT-3′		
*enlA*	for5′-TTCTTCTTATTCTGTCAACGCAGC-3′	960	Bittencourt de Marques and Suzart 2004
	rev5′-GACTGTGAAATACCTATTTGCAAGC-3′		
*esp*	for5′-TTGCTAATGCTAGTCCACGACC-3′	933	Shankar *et al*. 1999
	rev5′-GCGTCAACACTTGCATTGCCGAA-3′		
**Bacteriocin genes**			
*as*-48	for5′-GAGGAGGAGTATCATGGTTAAAG-3′	340	Sabia et al. 2008
	rev5′-CATATTGTTAAATTACCAAGCAATAA-3′		
*bac*31	for5′-GAAAAAGAAATTAGTTATTTGTG-3′	202	Sabia et al. 2008
	rev5′-CTATCTAGGAGCCCAAGG-3		
*entA*	for5′-ATGAAACATTTAAAAATTTTGTCTA-3′	130	Sabia et al. 2008
	rev5′-TTAGCACTTCCCTGGAATTG-3′		
*entB*	for5′-GAAAATGATCACAGAATGCCTA-3′	160	Du Toit *et al*. 2000
	rev5′-GTTGCATTTAGAGTATACATTTG-3′		
*entP*	for5′-GTTAGTTTGTGACAAATTTTGG-3′	120	Sabia et al. 2008
	rev5′-GGTGTGGAAAAGCCGTTTC-3′		
*entL50* A/B	for5′-TGGGAGAATCGCAAAATTAG-3′	96	Du Toit *et al*. 2000
	rev5′-ATTGCCCATCCTTCTCCAAT-3′		
*ent1071*	for5′-ATATTTAGGGGGACCGATAA-3′	405	Sabia et al. 2008
	rev5′-TATACATTCTTCCACTTATTTTT-3′		

The reactions conditions for each gene were the following:

For the amplification of the virulence genes *cylA, aggA, efaA, eep, gelE* and *esp,* the samples with the DNA templates were denatured at 94°C by 5 m. Subsequently, 30 cycles were performed: 45 s at 94°C, 1 m at 57°C and 1 m at 72°C for the gene *cylA;* 30 cycles of 45 s at 94°C, 1 m at 52°C and 1 m at 72°C for the *aggA* and *efaA* genes; 30 cycles of 45 s a 94°C, 1 m at 58°C and 1 m at 72°C for the *eep* gene; 30 cycles of 30 s at 94°C, 30 s at 56°C and 30 s 72°C for the *gelE* gene; 30 cycles of 45 s at 94°C, 1 m at 62°C and 1 m at 72°C for the *esp* gene*.* The amplification of all the virulence genes was concluded with a final extension of 72°C by 3 m.

The PCR were performed in a thermal DNA Engine (Bio-Rad, Hercules, CA). The negative controls (without DNA template) were included in each reaction set and all performed double. The amplification products were analyzed through electrophoresis in agarose gels at 1.5% stained with ethidium bromide and visualized under ultraviolet light. The size of each amplified product was confirmed by comparison with the molecular size marker 1Kb-Plus DNA ladder (New England Biolabs).

### Statistical analysis

The correlation between the presence of virulence and bacteriocin genes with the antimicrobial susceptibility of the *E. faecalis* strains, was determined using the Test of χ2 of Test Exact of Fisher, as it corresponded. A p < 0.05 value was considered statistically significant to indicate the association between both variables. The statistical package used was SPSS v.19.0.

## Results

From 128 water wells were isoalted 78 *E. faecalis* strains and were identified by conventional biochemical test.

### Virulence and bacteriocin PCR products

In the Table 2 it was observed that the *entA* gene (64.1%) is the most frequently detected followed by the *entL50A/B* (37.5%) and the *enlA* (26.9%) gene respectively. The *ent P* (8.9%) gene was the less frequent. The gene *as-48* was not detected. In the Table 3, it is observed that 11 of the total of the studied strains exhibit 6 virulence genes and 2 bacteriocin genes. The main genetic profile was observed in 18 *E. faecalis* strains*,* in which 4 different virulence genes and 2 bacteriocinogenic genes were detected. The less common profile corresponded to 5 strains that carried 3 virulence genes and 2 bacteriocinogenic genes. Eight genetic profiles were determined that include virulence and bacteriocin genes (A, B, C, D, E, F, G y H profiles).

**Table 2:** **Virulence and bacteriocin genes in 78*Enterococcus faecalis*strains isolated from water wells for human consumption**

Virulence genes	Nº	%
*aggA*	73	93.5
*cylA*	60	76.9
*efaA*	37	47.4
*eep*	31	39.7
*esp*	55	70.5
*gelE*	24	30.7
Bacteriocin genes		
*entB*	13	16.6
*ent 1071*	8	10.2
*ent P*	7	8.9
*entL50A/B*	29	37.5
*enlA*	21	26.9
*entA*	50	64.1
*bac31*	11	14.1
*as-48*	0	0

**Table 3:** **Virulence and bacteriocin genetic profiles in 78*E. faecalis*strains isolated from water wells for human consumption**

Genetic profiles		Strains	
**Virulence genes**	**Bacteriocins**	**nº**	**%**
(A) * *efaA, aggA, esp, gelE, cylA, eep*	*entA, bac31*	11	14.1
(B) *efaA, aggA, esp, gelE, cylA*	*entA, entB*	8	10.2
(C) *efaA, aggA, esp, gelE*	*entA*, *entL50A/B*	18	23.0
(D) *aggA, esp, cylA, eep*	*enlA, entP*	7	8.9
(E) *aggA, esp, cylA*	*entL50A/B, enlA*	11	14.1
(F) *cylA, gelE, eep*	*entA, entB*	5	6.4
(G) *cylA, eep, aggA*	*entA, ent 1071*	8	10.4
(H) *cylA, aggA, gelE*	*entA, enlA*	10	12.8
Total		78	100

*: Name of genetic profile.

### Antimicrobial susceptibility

The Table 4 shows the resistance phenotypes (MICs) of the 78 *E*. *faecalis* strains. It is observed that 3 phenotypes exhibit resistance to 4 antibiotics simultaneously and 3 phenotypes show resistance to only one. In the same table, it is observed that the most common resistance phenotype (n = 14) corresponds to a tetracycline and chloramphenicol and the less frequent (n = 2) to ciprofloxacin and moxifloxacin. It was not observed resistance to vancomycin in any of the strains evaluated.

**Table 4:** **MICs of several antimicrobials against different resistance phenotypes of*E. faecalis*isolated from wells of water for human consumption**

Median MIC (range) (mg/L)									
**Resistance phenotype**	**Numbers of strains**	**T**	**A**	**V**	**C**	**M**	**Ch**	**G**	**E**
T A Ch G	8	64 (16–256)	16 (4–128)				64 (32–256)	16 (8–128)	
G A Ch E	10		16 (8–64)				64 (32–128)	32 (16–64)	16 (8–64)
T A Ch E	7	32 (8–128)	8 (8–64)				16 (8–128)		16 (8–64)
T G A	11	64 (16–128)	8 (8–64)					32 (16–128)	
T Ch	14	64 (16–256)					64 (32–256)		
A G	6		16 (8–128)					32 (16–256)	
T C	4	32 (8–128)			8 (4–32)				
C M	2				8 (4–32)	16 (32–128)			
Ch	5						64 (32–256)		
T	6	64 (16–128)							
G	5							32 (16–256)	
Total	78								

T: tetracycline; A: ampicillin; V: vancomycin ; C: ciprofloxacin; M: moxifloxacin; Ch: chloramphenicol; G: gentamicin; E: erythromicin.

### Relation between antimicrobial susceptibility and genetic profiles

In accordance to statistical analysis it was observed a significant association between the resistance to tetracycline and chloramphenicol with the genetic profiles A, B, C, D and F and of sensitivity in presence of the genetic profiles E and H. It was observed that ampicillin resistance is associated to the genetic profiles A, B, C y D and of sensitivity with E and F (p < 0.0001). The genetic profiles associated with the resistance to gentamicin were A, B, D and F and of sensitivity C and E (p < 0.0001). Erythromicin, ciprofloxacin, moxifloxacin and vancomycin did not exhibit a significant statistically susceptibility association with the genetic profiles detected.

In the Table 5, it is observed that the *E. faecalis* strains that exhibit a greater number of virulence and bacteriocin genes were more resistant to the antimicrobial. The greatest number of resistant strains was associated with tetracycline followed by chloramphenicol, ampicillin and gentamicin respectively. The lowest resistance of the strains was observed for moxifloxacin and ciprofloxacin.

**Table 5:** **Relation of virulence and bacteriocin genetic profiles and their respective antimicrobial resistance in*E. faecalis*strains isolated from water wells for human consumption**

Virulence and bacteriocin		Nº of resistant strains and antimicrobials genetic profiles							
		T	A	V	C	M	Ch	G	E
*efaA, aggA, esp, gelE, cylA, eep*	*entA, bac31*	11	9	0	2	1	11	8	3
*efaA, aggA, esp, gelE, cylA*	*entA, entB*	6	8	0	1	0	8	8	4
*efaA, aggA, esp, gelE*	*entA*, *entL50A/B*	12	11	0	1	1	11	3	3
*aggA, esp, cylA, eep*	*enlA, entP*	5	4	0	0	0	5	7	2
*aggA, esp, cylA*	*entL50A/B, enlA*	3	3	0	0	0	0	4	1
*cylA, gelE, eep*	*entA, entB*	3	1	0	0	0	3	4	1
*cylA, eep, aggA*	*entA, ent 1071*	4	2	0	0	0	4	2	0
*cylA, aggA, gelE*	*entA, enlA*	5	2	0	0	0	2	3	3
Total		49	40	0	4	2	44	39	17

T: tetracycline; A: ampicillin; V: vancomycin ; C: ciprofloxacin; M: moxifloxacin; Ch: chloramphenicol; G: gentamicin; E: erythromicin.

## Discussion

*Enterococcus faecalis* is a very important bacterial specie as a pathogen acquired in hospitals of USA and Europe, especially in intensive care units, where are one of the main causes of bacteremia (Rodríguez-Baño *et al.*^2005^). Although there are various surveys addressed to know the causes because these microorganisms induce human infections, diverse aspects of its biology have not been revealed (Fisher and Phillips 2009).

The results demonstrated that the majority of the bacterial strains had genes that codify for different virulence factors. The results of this study are coherent with the described in other studies (Koch *et al.*^2004^; Dupont *et al*. 2008). The enterocin genes analyzed in this work represent the most frequently described in this bacterial specie (Franz *et al.*^2007^). The bateriocinogenic activity of *E. faecalis* is widely recognized and its bacteriocins have been object of interesting biotechnological studies associated to the biopreservation of foods (Khan *et al*. 2010; Poeta *et al.*^2007^). The bacteriocinogenic capacity of these microorganisms grants an ecological superiority with respect to those strains that do not produce these antibacterial peptides. This property also grants survival advantages to the bacteriocinogenic strains, because these products are able to eliminate to potential competing microorganisms and start subsequently the colonization phase. It was very interesting to observe that all the studied strains exhibited two different types of genes for bacteriocins, which added to its respective virulence genes grant to these strains a greater pathogenic potentiality. Thus, bacteriocinogenic strains have an additional ecological tool to persist in environments colonized by competing microorganisms. Probably once introduced in the organism with the water these strains can more easily colonize; on the contrary they do not have an ecological benefit in aquatic environment where virulence factors and bacteriocins are diluted.

It was shown in this finding that most of the strains exhibit resistance to two or more antimicrobials. Specifically 25 strains present resistance to four different types of the antimicrobials used. This important resistance associated to high MICs confirms the recognized antimicrobial property of *E. faecalis* which is maintained in the studied strains. Also, in accordance to statistical analysis it is possible to argue that genetic profiles A, B, and D of *E. faecalis* are more frequently associated to resistance against some antimicrobials and inversely, the profiles C and E are associated with sensitivity.

The evolution and ecology of *E. faecalis*, is a subject of great importance and that must be constantly monitored to have new information to allow to know the biology of this bacterial specie. The results of this study demonstrate that intensive use of water wells for human consumption includes poor sanitary practices. This is the cause of bacterial contamination of the wells with human microbiota. This could explain the presence of *E. faecalis* in these wells. Although, generally contamination of the wells is due to environmental contamination of groundwater by fecal pollution, it is possible to consider that *E. faecalis* strains analyzed are circulating among the wells and the people who use this water resource. The observed properties in the studied bacterial strains are similar to those found in strains isolated from clinical samples. On the other hand, these strains are a serious problem for people who drink this water contaminated with *E. faecalis*, considering that these bacteria could transfer genetic information from antimicrobial resistance to their intestinal microbiota (De Niederhäusern *et al*. 2011).

## References

[CR1_86] Arthur M Molina C Depardieu F Courvalin P Characterization of TN1546, a Tn3 – related transposon conferring glycopeptide resistance by synthesis of depsipeptide peptidoglycan precursors in Enterococcus faecium BM4147 J Bacteriol 1993 175 117 127 8380148 10.1128/jb.175.1.117-127.1993 PMC196104

[CR2_86] Bauer A Kirby M Sherris J Turck M Antibiotic susceptibility testing by a standardized single disk method Amer J Clin Pathol 1996 45 493 496 5325707

[CR3_86] Biendenbach DJ Moet GJ Jones RN Occurrence and antimicrobial resistance pattern comparisons among bloodstream infection isolates from the SENTRY antimicrobial surveillance program Diag Micr Infec Dis 2004 50 59 69 10.1016/j.diagmicrobio.2004.05.003 15380279

[CR4_86] Bittencourt de Marques E Suzart S Occurrence of virulence- associated genes in clinical Enterococcus faecalis strains isolated in Londrina, Brazil J Med Microbiol 2004 53 1069 1073 10.1099/jmm.0.45654-0 15496382

[CR5_86] Chang CM Wang LR Lee HC Lee NY Wu CJ Ko WC Characterization of vancomycin-resistant enterococci from hospitalized patients at a tertiary center over a seven-year period J Hosp Infect 2010 74 377 384 10.1016/j.jhin.2009.10.025 20170984

[CR6_86] Clewell DB Flannagan SE Jaworski DD Unconstrained bacterial promiscuity: the Tn916-Tn1545 family of conjugative transposons Trends Microbiol 1995 3 229 236 10.1016/S0966-842X(00)88930-1 7648031

[CR7_86] Cookson BD Macrae MB Barret SP Brown DJ Chadwick C French GL Hateley P Hosein IK Guidelines for the control of glycopeptide-resistant enterococci in hospitals J Hosp Infect 2006 62 6 21 10.1016/j.jhin.2005.02.016 16310890

[CR8_86] De Niederhäusern S Bondi M Messi P Iseppi R Sabia C Manicardi G Anacarso I Vancomycin-resistance transferability from VanA enterococci to Staphylococcus aureus Curr Microbiol 2011 62 1363 1367 10.1007/s00284-011-9868-6 21234755

[CR9_86] Du Toit M Franz CM Dicks LM Holzapfel WH Preliminary characterization of bacteriocins produced by Enterococcus faecium and Enterococcus faecalis isolated from pig faeces J Appl Microbiol 2000 88 482 494 10.1046/j.1365-2672.2000.00986.x 10747229

[CR10_86] Dupont H Vael C Muller-Serieys C Chosidow D Marmuse J Andremont A Goossens H Desmonts J Prospective evaluation of virulence factors of enterococci isolated from patients with peritonitis. Impact on outcome Diag Microb Infect Dis 2008 60 247 253 10.1016/j.diagmicrobio.2007.10.006 18060725

[CR11_86] EUCAST disk diffusion method for antimicrobial susceptibility testing 2012

[CR12_86] Facklam R Collins M Identification of Enterococcus species isolated from human infections by a conventional test scheme J Clin Microbiol 1989 27 731 734 2656745 10.1128/jcm.27.4.731-734.1989 PMC267406

[CR13_86] Fisher K Phillips C The ecology, epidemiology and virulence of Enterococcus Microbiology 2009 155 1749 1757 10.1099/mic.0.026385-0 19383684

[CR14_86] Franz CM Van Belkum MJ Holzapfel WH Abriouel H Gonçalvez A Diversity of enterococcal bacteriocins and their grouping in a new classification scheme FEMS Microbiol Rev 2007 31 293 310 10.1111/j.1574-6976.2007.00064.x 17298586

[CR15_86] Giraffa G Enterococcal bacteriocins: their potential use as anti-Listeria factors in dairy technology Food Microbiol 1995 12 551 556

[CR16_86] Hidron AI Edwards JR Patel J Hora TC Sievert DM Pollock DA Fridkin SA NHSN annual update: antimicrobial-resistant pathogenes associated with heathcare-associated infections: annual summary of data reported to the National Healthcare Safety Network at the Centers for Disease Control Prevention, 2006–2007 Infect Control Hosp Epidemiol 2008 29 996 1011 10.1086/591861 18947320

[CR17_86] Khan H Flint S Yu PL Enterocins in food preservation Int J Food Microbiol 2010 141 1 10 10.1016/j.ijfoodmicro.2010.03.005 20399522

[CR18_86] Koch S Hufnagel M Theilackers C Huebner J Enterococcal infections host response therapeutic and prophylactic possibilities Vaccine 2004 22 822 830 10.1016/j.vaccine.2003.11.027 15040934

[CR19_86] Köhler W The present state of species within the genera Streptococcus and Enterococcus IJMM 2007 297 133 150 17400023 10.1016/j.ijmm.2006.11.008

[CR20_86] Martínez-Odrizola P Muñoz-Sánchez P Gutierrez-Macías A Arriola-Martínez P Montero-Aparicio E Ezpeleta-Baquedano C Análisis de 182 episodios de bacteremia por enterococo: estudio de la epidemiología, microbiología y evolución clínica Enferm Infecc Microbiol Clin 2007 25 503 507 10.1157/13109986 17915108

[CR21_86] Naser S Thompson FL Hoste B Gevers D Vandmeulebroecke K Cleenwerck I Thompson C Vancanneyt M Phylogeny and identification of enterococci by atpA genee sequence analysys J Clin Microbiol 2005 43 2224 2230 10.1128/JCM.43.5.2224-2230.2005 15872246 PMC1153757

[CR22_86] Padilla C Lobos O Brevis P Abaca P Hubert E Plasmid-mediated bacteriocin production by Shigella flexneri isolated from dysenteriae diarrhea and their transformation into Escherichia coli Lett Appl Microbiol 2006 42 300 303 10.1111/j.1472-765X.2005.01829.x 16478521

[CR23_86] Poeta P Costa D Rojo-Bezares B Zarazaga M Klibi N Rodriguez J Torres C Detection of antimicrobial activities and bacteriocin structural genes in faecal enterococci of wild animals Microbiol Res 2007 162 257 263 10.1016/j.micres.2006.06.003 16872815

[CR24_86] Poh CH Oh HM Tan AL Epidemiology and clinical outcome of enterococcal bacteraemia in an acute care hospital J Infect 2006 52 383 386 10.1016/j.jinf.2005.07.011 16203039

[CR25_86] Ridwan B Mascini E Van Der Reijden N Verhoef J Bonten M What action should be taken to prevent spread of vancomycin resistant enterococci in European hospitals? BMJ 2002 324 666 668 10.1136/bmj.324.7338.666 11895829 PMC1122583

[CR26_86] Rodríguez-Baño J Ramírez E Muniain M Santos J Joyanes P González F García-Sánchez M Martínez-Martínes L Colonization by high-level aminoglycoside-resistant enterococci in intensive care unit patients. Epidemiology and clinical relevance J Hosp Infect 2005 60 353 359 10.1016/j.jhin.2004.12.024 15893852

[CR27_86] Sabia C Niederhaüsen S Guerrieri E Anacarso I Manicardi G Bondi M Detection of bacteriocin production and virulence traits in vancomicyn-resistant enterococci of different sources J Appl Microbiol 2008 104 970 979 10.1111/j.1365-2672.2007.03612.x 18005029

[CR28_86] Semedo T Santos MA Lopes MF Marquez JJ Crespo MT Tenreiro R Virulence factors in food, clinical and reference enterococci: a common trait in the genes? Syst Appl Microbial 2003 26 13 22 10.1078/072320203322337263 12747405

[CR29_86] Shankar V Baghdayan AS Huycke MM Lindahl G Gilmore MS Infection-derived Enterococcus faecalis strains are enriched in esp, a gene encoding a novel surface protein Infect Immun 1999 67 193 200 9864215 10.1128/iai.67.1.193-200.1999 PMC96296

[CR30_86] Shepard BD Gilmore MS Differential expression of virulence related genes in Enterocococcus faecalis in response to biological cues in serum and urine Infect Immun 2000 70 4344 4352 10.1128/IAI.70.8.4344-4352.2002 12117944 PMC128128

[CR31_86] Treitman AN Yarnols PR Warren J Noskin GA Emerging incidence of Enterococcus faecium among hospital isolates (1993 to 2002) J Clin Microbiol 2005 43 462 463 10.1128/JCM.43.1.462-463.2005 15635016 PMC540171

[CR32_86] Eaton TJ Gasson MJ Molecular screening of Enterococcus virulence determinants and potential for genetic exchange between food and medical isolates Appl Environ Microbiol 2001 67 1628 1635 10.1128/AEM.67.4.1628-1635.2001 11282615 PMC92779

